# Investigating the association between photosynthetic efficiency and generation of biophotoelectricity in autotrophic microbial fuel cells

**DOI:** 10.1038/srep31193

**Published:** 2016-08-09

**Authors:** Gustavo P. M. K. Ciniciato, Fong-Lee Ng, Siew-Moi Phang, Muhammad Musoddiq Jaafar, Adrian C. Fisher, Kamran Yunus, Vengadesh Periasamy

**Affiliations:** 1 Institute of Ocean and Earth Sciences (IOES), University of Malaya, 50603 Kuala Lumpur, Malaysia; 2 Department of Chemical Engineering and Biotechnology, University of Cambridge, New Museums Site, Pembroke Street, CB2 3RA Cambridge, United Kingdom; 3 Institute of Biological Sciences, Faculty of Science, University of Malaya, 50603 Kuala Lumpur, Malaysia; 4 Low Dimensional Materials Research Centre (LDMRC), Department of Physics, University of Malaya, 50603 Kuala Lumpur, Malaysia

## Abstract

Microbial fuel cells operating with autotrophic microorganisms are known as biophotovoltaic devices. It represents a great opportunity for environmentally-friendly power generation using the energy of the sunlight. The efficiency of electricity generation in this novel system is however low. This is partially reflected by the poor understanding of the bioelectrochemical mechanisms behind the electron transfer from these microorganisms to the electrode surface. In this work, we propose a combination of electrochemical and fluorescence techniques, giving emphasis to the pulse amplitude modulation fluorescence. The combination of these two techniques allow us to obtain information that can assist in understanding the electrical response obtained from the generation of electricity through the intrinsic properties related to the photosynthetic efficiency that can be obtained from the fluorescence emitted. These were achieved quantitatively by means of observed changes in four photosynthetic parameters with the bioanode generating electricity. These are the maximum quantum yield (Fv/Fm), alpha (α), light saturation coefficient (Ek) and maximum rate of electron transfer (rETRm). The relationship between the increases in the current density collected by the bioanode to the decrease of the rETRm values in the photosynthetic pathway for the two microorganisms was also discussed.

Microbial fuel cells (MFCs) are a novel and promising technology currently under development with the purpose of generating electricity through bioelectrochemical processes, by taking advantage of natural microbial redox activities^1^. The engineering of such devices are found in the literature with a large variety of designs that are developed to fit with the microorganisms’ needs, as well as contribute on improvements in their performance. These improvements are mainly focussed on increasing the power output through the decrease of losses such as Ohmic polarization resulting from high internal resistances, activation polarization resulting from the poor electrical contact between the microoganisms with the electrode surface, and concentration polarization resulting from the low availability of substrates^2^. The latter may result either from the poor transport of mediators or nutrients in solution, or oxygen from the atmosphere, in the case of devices using air-breathing cathodes^3^. A noteworthy type of MFC, conventionally designated as a biophotovoltaic (BPV) device, involves the use of photoautotrophic microorganisms, where the source of the electrons is suggested to be light-driven, having the generation of electricity not depending on the use of organic substrates^4^. Typical BPVs proposed in the literature consider the use of different sources of microbial biocatalysts such as algae^5^, cyanobacteria^6^, or even soil microorganisms associated with moss, and even plants^7^. Other bioelectrochemical devices comprising organelles, or other sub-cellular structures related to photosynthesis can be found in the literature, and referred to as BPVs, but in this paper, we only consider BPVs comprising whole, living microorganisms.

BPVs present unique advantages for the generation of true green and clean energy, such as having a negative or neutral carbon footprint though the removal of CO_2_ from the environment^8^, and the unlimited capacity of producing the biocatalysts, considering that the microorganisms can grow and reproduce for as long as the environmental conditions allow it^9^. However, their present efficiency remains considerably low. Recent efforts to increase the performance for generation of electricity in BPVs include the selection of strains with high photosynthetic efficiency^10^, the engineering of materials to improve the microenvironment necessary for the biofilm formation, and increase in the surface area for electron transfer from the microorganism to the electrode surface^11^. Similarly, the creation of metabolic mutants containing modifications in terminal oxidases can be used for the increase in the biocatalytic activity^12^. Part of the problem still resides in the lack of knowledge of the fundamental bioelectrochemical mechanisms behind the electron transfer between these microorganisms and the electrode surface. Most fundamental studies on this subject focused on bioelectrodes containing heterotrophic microorganisms for conventional MFCs, such as those from the genera *Geobacter*^13^, *Shewanella*^14^, and *Rhodoferax*^15^. Few studies have been carried out for bioelectrodes comprising autotrophs.

It was suggested that electricity generated by the bioanode in a BPV could have direct association with the electrons involved within the photosynthetic pathway^16^. Piscotta *et al*.^17^ used the Cyanophytes *Lyngbya* and *Nostoc* species in a photosynthetic microbial fuel cell (PMFC) and observed that the electrons originated from water photolysed by photosystem II (PSII) and that the transfer of these electrons to extracellular electron acceptors was mediated by plastoquinone and cytochrome bd quinol oxidase. This relationship was strengthened with the observation that the lack of photosystem II (PSII) in the *Synechocystis* adsorbed on a bioanode strongly affected the amount of electrical current generated, in comparison to the same containing PSII^18^. Complementary information related to the photosynthetic efficiency can be obtained through the fluorescence response these microorganisms present in consequence to the absorption of light. The use of the pulse amplitude modulation fluorescence (PAM) has become a very popular tool to examine photosynthetic materials, and assess their photosynthetic efficiency^19^. Important parameters associated with the efficiency of capture and absorption of light through chlorophyll-a (Chl-a) and other antennae pigments, as well as the kinetics and efficiency of PSII, PSI, and the subsequent electron carriers within the transfer of electrons in the photosynthetic pathway can be measured^20^.

In this work, we propose the utilization of the PAM as an analytical technique to probe the effect of the generation of electricity from bioelectrodes containing photoautotrophic microorganisms on their photosynthetic efficiency. We believe that the information obtained from this current work will greatly contribute to the understanding and development of BPVs in future.

## Results

### F_v_/F_m_ the maximum quantum yield

One of the most important parameters in photosynthesis, and related to its photosynthetic efficiency is the maximum quantum yield, commonly known as the ratio F_v_/F_m_. This parameter expresses the maximum light utilization efficiency (absorbed photons are converted to electron flow) measured in the dark^21^. The measurement of F_v_/F_m_ involves the first step of complete dark adaptation of the biofilm in the electrochemical device. A period of 15 minutes is conventionally used for the dark adaptation as a proxy method of estimating other important parameters, even though some residual level of non-photochemical quenching can still be observed in some cases^22^. The dark adaptation is established when there are no photons striking the antennae complexes, and the reaction centers (both PSII and PSI) are said to be in the oxidized form. These reaction centers are conventionally described as “open” after the dark adaptation, and are now ready to accept excitation energy from light source as well as other antenna. Besides that, some background fluorescence can still be measured, and this minimum fluorescence is defined as F_0_. By the application of a high pulse of light whose intensity depends on the nature of the photosynthetic material, it is possible to activate all the reaction centers, promoting the removal of electrons from the PSII to the photosynthetic chain^23^. The active centers, being available to donate electrons to the next electron acceptors are now considered to be “closed”. Therefore, the efficiency with which the photosynthetic material transform from the state where all the reaction centers are open (minimum fluorescence, F_0_) to the state where all the reaction centers are closed (maximum fluorescence, F_m_), is described by the maximum quantum yield.

**Figure 1:**
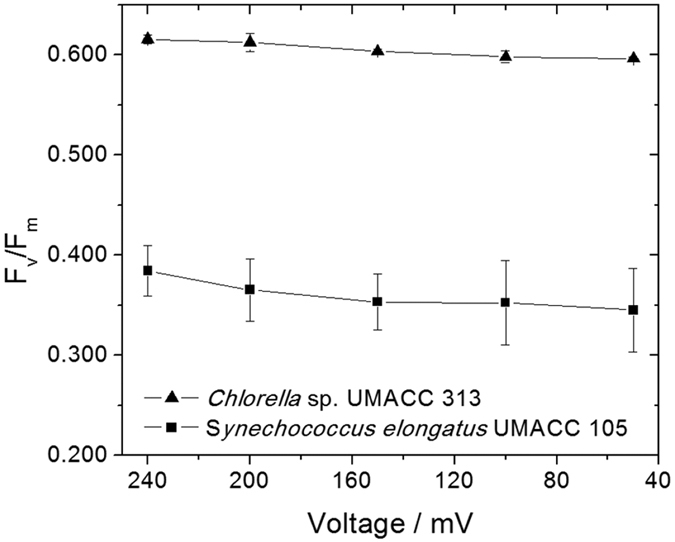
Effect of the electrochemical cell voltage to the maximum quantum yield, F_v_/F_m_ in biofilms of *Chlorella* and *Synechococcus* as bioanodes connected in the electrochemical device.

Figure 1 presents the values of F_v_/F_m_ measured for the biofilms of both *Chlorella*, and the *Synechococcus* formed on the top of the ITO as a bioanode operating in an electrochemical cell under conditions of OCP, and with the variation of the electrochemical cell voltage. At the conditions of OCP, when both bioanode and cathode are not connected, and electrical current is not flowing, the values of F_v_/F_m_ for the biofilm with *Chlorella* and *Synechococcus* were 0.62 ± 0.04 and 0.38 ± 0.03, respectively. Since the biofilms were formed with the two microorganisms growing under its optimum conditions of medium concentration, temperature and light conditions, the parameters obtained at OCP are considered to be related to its healthy (non-harmed) condition.

The addition of a resistor in the circuit brings about a decrease in the electrochemical cell voltage, and then electrical current starts to flow from the bioanode to the cathode through bioelectrochemical reactions^10^. The observed effect to the flow of electrical current is indicated by the decrease in the values of F_v_/F_m_ in both cases. Values of F_v_/F_m_ for the electrochemical cell running at voltage of 50 mV for the biofilm with *Chlorella* (0.60 ± 0.01) and *Synechococcus* (0.35 ± 0.04) were significantly different (p < 0.05) (Table 1). This represents a decrease of 4 and 11% when compared to the same conditions of OCP. As such, the result suggests that *Synechococcus* is more susceptible to the effect of the voltage applied than *Chlorella*. This is interesting as the *Synechococcus* biofilm contained higher biomass ([Chl-a] = 1920 mg.m^−3^) than the *Chlorella* biofilm ([Chl-a] = 715 mg.m^−3^).

### The kinetics parameters E_k_ and alpha (α)

Through the construction of rapid light curves or RLCs, it is possible to obtain important parameters related to the light utilization by the photosynthetic material. RLCs typically show a quasi-linear, light-limited increase in photosynthetic rate under low values of irradiance, in terms of photosynthetically available photon flux density, before reaching a photosynthetic value at a maximum light-saturated rate (P_max_). Under conditions of excessive high irradiance, after crossing the maximum light-saturated rate, the photosynthetic value tends to decrease due to the conditions of excess light that can be harmful to the photosynthetic apparatus, generally as by the inactivation of PSII^24^, or inhibition of the enzyme Ribose-1,5-biphosphate carboxylase/oxygenase (RuBisCo) caused by heat stress^25^.

The first kinetics parameter to be obtained is the slope of the initial linear, light-limited part of the light curve, termed alpha (α), and the second is the light saturation coefficient, or sometimes referred to as light adaptation E_k_. A third parameter can be considered which is related to the appearance of a second negative slope related to the decrease in the photosynthetic rate or inactivation of the photosynthetic material under conditions of high illumination, termed beta (β). Since the experiments performed were kept outside of this region of irradiance to avoid permanent damage to the cells, this parameter was not considered for this work.

**Figure 2:**
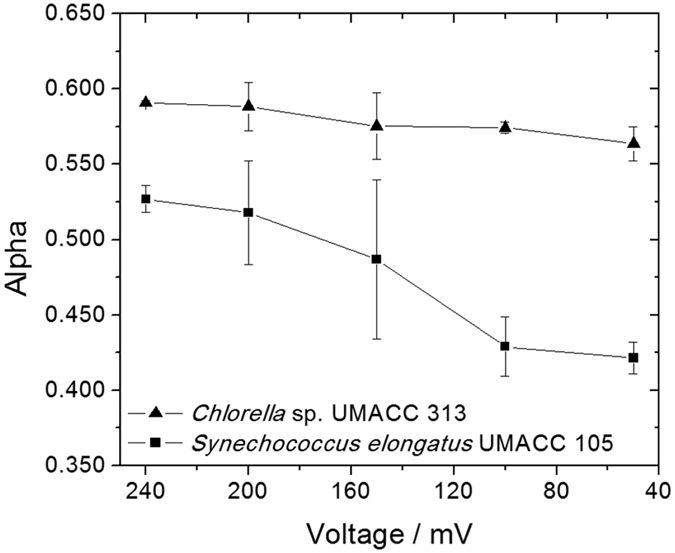
Effect of the electrochemical cell voltage to alpha (α) in biofilms of *Chlorella* and *Synechococcus* as bioanodes connected in the electrochemical device.

Figure 2 shows the values of alpha (α) obtained for the RLCs with the biofilm grown on ITO, and connected as an anode to the electrochemical cell under conditions of OCP, and under the application of an external voltage to have the circulation of current.

From Fig. 2 it is possible to observe that the values of alpha (α) for both microorganisms decreased under conditions of circulation of electrical current. The values of alpha (α) obtained for the bioanode containing biofilms of *Chlorella* and *Synechococcus* were, 0.59 ± 0.01 and 0.53 ± 0.01, respectively. Under conditions of OCP, the values for both biofilms were 0.56 ± 0.01 and 0.42 ± 0.01 measured at 50 mV, respectively.

The next parameter that should be looked at is the parameter E_k_, which is a correlation for the parameter alpha (α) with the maximum light-saturated rate (P_max_). Different from the observations in Figs 1 and 2, the changes for E_k_ in both cases do not seem to be very consistent. The values of E_k_ with the voltage in the electrochemical cell for the bioanode prepared for both *Chlorella* and *Synechococcus* do not appear to be linear. In fact, the high distributions of values obtained led to a considerably high standard deviation (See Supplementary Figs S1 and S2).

### The maximum relative electron transfer rate, rETR_m_

Finally, the most important parameter considered for this work is the rETR_m_, which represents the empirical estimate of the maximum rate of flow of electrons through the electron flow pathway. First presented by Genty *et al*.^26^, the calculation of rETR_m_ is based primarily on the assumption that the photon yield is given by the product of the efficiency of an open PSII reaction center and the fraction of open reaction centers^26^. Considering that it is necessary one single photon for the removal of every single electron from PSII, the overall rate of electron flow can be calculated by multiplying the photon yield with the amount of photons absorbed by PSII. This calculation takes into consideration that the slowest step in the electron transport chain is the re-oxidation of the plastoquinone acceptor Q_B_. In practice, the carboxylation or a step closely associated to it is the overall rate-limiting step under normal light conditions^27^. To guarantee that the experiments are performed in such a way so as to avoid these conditions, the sample is exposed to short (μ-second) pulses of light through the modulated measuring beam with a relatively long lag between the pulses, which will only induce fluorescence but not photochemistry^28^.

**Figure 3:**
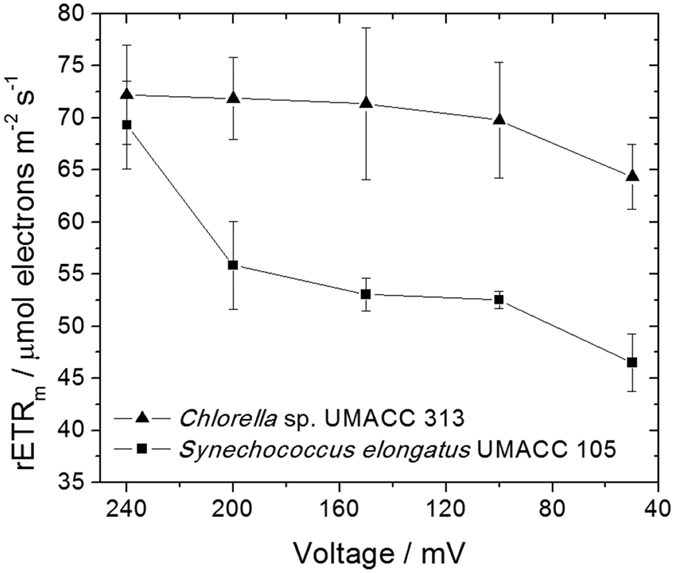
Effect of the electrochemical cell voltage to the maximum of electron transfer rate, rETR_m_ in biofilms of *Chlorella* and *Synechococcus* as bioanodes.

Figure 3 demonstrates the maximum electron transfer rate measured for the two microorganisms in a biofilm on ITO connected to the electrochemical cell under conditions of OCP and with decrease of load.

The values of rETR_m_ measured for the bioanodes containing biofilms of *Chlorella* and *Synechococcus* under conditions of OCP were 72.23 ± 4.75 and 69.35 ± 4.22 μmol electrons.m^−2^.s^−1^, respectively while the same under the electrochemical cell running at 50 mV were 64.37 ± 3.12 and 46.49 ± 2.75 μmol electrons.m^−2^.s^−1^, respectively. This represents a decrease in the values of relative electron transfer rate of 11% for *Chlorella* and a significant decrease of 33% (p < 0.05) for *Synechococcus*.

**Figure 4:**
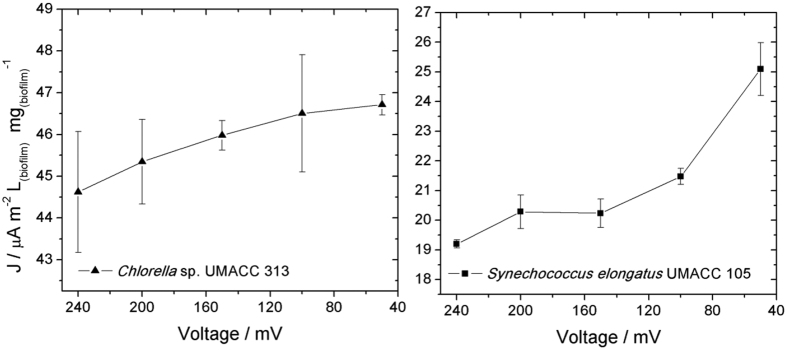
Polarization curves of the electrochemical cell in biofilms of *Chlorella* and *Synechococcus* as bioanodes.

With an attempt to evaluate the rETR_m_ with the BPV performance, it is necessary to verify the polarization curves resulting from these experiments, presented in Fig. 4.

It is observed from Fig. 4, an increase in the current density with the decrease of the electrochemical cell voltage in both cases, as expected for a galvanic cell. Extra care was taken with the analysis of the results presented in Fig. 4 due to the fact that the experiment was not performed in conditions of steady state (from the point of view of the electrochemical measurements), resulting in the considerably high values of current density caused by non-faradaic processes.

**Figure 5:**
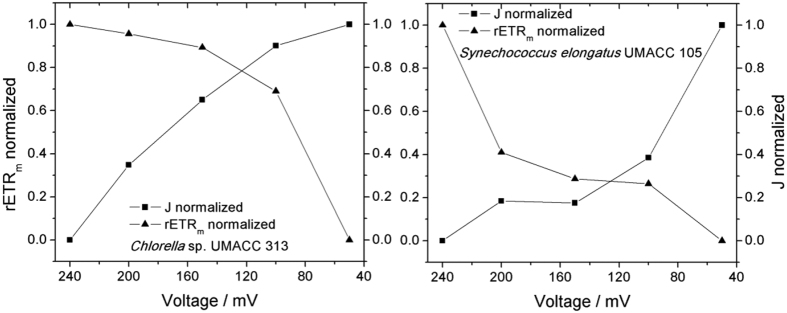
Normalized curves of current and maximum rate of electron transfer obtained by the electrochemical cell in biofilms of *Chlorella* and *Synechococcus* as bioanodes.

Since the time scale was chosen to fit the experiments involving the fluorescence measurements, but not necessarily the electrochemical ones, a more accurate way to discuss the results presented in Figs 3 and 4 is through normalization of both curves, as presented in Fig. 5.

Simple statistical analysis gives a correlation coefficient of −0.76 and −0.79 for the values of current density and rETR_m_ for *Chlorella* and *Synechococcus* bioelectrodes, respectively.

## Discussion

The F_v_/F_m_ is typically used to measure the stress conditions that the photosynthetic apparatus or more specifically, the PSII and its electron carriers Q_A_ and Q_B_, are experiencing. It was observed previously that environmental conditions such as drought^29^, heat^30^, and nutrient limitation resulting from the lack or simply accessibility of nutrients^31^, can affect the values of F_v_/F_m_ when compared to the same system in a non-stressful and healthy conditions.

The experiments performed in this work were executed in the same conditions of biofilm growth, nutrients availability and light exposure. As such, the only parameters that may be causing stress to the cells structure should have electrical origin. The decrease in the values of F_v_/F_m_ observed is quasi-linear in relation to the decrease in the electrochemical cell voltage for both microorganisms. It is important to point out that the application of the voltage to the electrochemical cell is accompanied not only by the flow of current responsible for the production of electricity, but also by the generation of an electric field. This electric field can interact and modify the proteins and lipids that constitute cellular structures and organelles, and affect photosynthesis directly or indirectly. It is known that application of an electrical field to the cell wall may cause weakening of the lipid-lipid interactions in the lipid bilayer membrane^32^. In extreme cases, the effect of strong electrical fields in the order of kV.cm^−1^ can exceed the dielectric strength of the cell membrane, and result in the formation of hydrophobic pores through a process called electroporation, increasing the permeability of the lipid bilayer, or in some cases resulting in its disruption^33^. The increase in the flow of electrical current per unitary cell, even with a considerably low value, can also cause the increase of temperature locally, resulting in additional stress that can affect not only the cell itself, but the biofilm as a whole. The stress on the microorganisms resulting from the generation of electricity as a bioanode does not seem to be permanent. Independent to the source of this stress, the original values of F_v_/F_m_ could be easily recovered by moving the electrochemical cell with the bioelectrodes containing the microorganisms back to the incubator for 15 minutes

The value of alpha (α) calculated through the slope in the linear region of the light curve is related to the light requirements for the microorganism to reach its maximum photosynthetic activity. With the increase of voltage applied from 50 to 240 mv, the α values were observed to increase in *Chlorella* sp. (α increased by 4.8%) and *Synechococcus* (α increased by 25.2%). The photosynthetic efficiency of the algae may have been enhanced by the increased voltage, as exhibited by the increased α values.

The photosynthetic parameter E_k_ represents the value of light intensity in which the photosynthetic rate of the studied material is optimal, and the light absorbed by the active centers equals the rETR_m_ or maximum electron transport rate^34^. It also gives the level of photoadaptation that indicates the threshold of light exposure that can affect its health and growth. Garcia-Mendoza^35^ showed that growing the Chlorophyte *Chlorella fusca* under light conditions with irradiance lower than E_k_ presented a situation where the microorganism can easily cope with their light environment, and adapt itself for an optimum growth while situations under light conditions with irradiance higher than E_k_ would reduce its growth potential^35^. Although changes associated with differential photo acclimation exist, these changes are considered to be developmental, i.e. controlled by gene expression^36^. Such changes involving adaptation by protein and pigment synthesis take more than 30 minutes, typically several hours or even several days^37^. Since the time required to perform each experiment was less than 30 minutes, such changes related to photo-adaptation is not expected to affect the experiments. Although the biofilm was always brought into incubation to recover from any possible stress caused by the experiment itself, the possibility of having modifications in some important structures responsible for the gene expression apparatus resulting from the applied voltage, cannot be disregarded. Since the time scale for the performed experiments is too short to evoke such effects, further experiments are necessary to analyze the effects of long term adaptation to the microorganisms present in the biofilms in terms of E_k_ as well as genomic modifications.

For the electrochemical measurements, it is possible to observe that similar to the observations in Fig. 1, the photosynthetic kinetics involved in *Chlorella* appears to be less influenced by the circulation of electrical current in its biofilm as a bioanode in comparison with *Synechococcus*. Under both conditions of OCP, and with circulation of electrical current, the bioelectrode containing *Chlorella* behaved with fast photosynthetic kinetics. On the other hand, the bioelectrode containing *Synechococcus* showed a complete change in its photosynthetic behavior, changing from a fast kinetics under conditions of OCP to a low kinetics with circulation of electrical current.

The observed effect of having the circulation of electrical current in the electrochemical device is the decrease in the photosynthetic parameter rETR_m_. This time, the circulation of current in the bioanode provokes a “slowdown” to the relative rate of electron transfer happening in the chain of electrochemical reactions involving the transport of charges from photosystem II to photosystem I. Two possible phenomena may be responsible for the decrease in the rETR_m_. First, some of the chemicals responsible for the functionality of the photosystems may have been affected by the electrical or electrochemical environment that the biofilm was experiencing, following the changes observed in F_v_/F_m_. Second, the electrons that have been collected by the bioanode during the generation of electricity can be linked to one of the redox components acting in this chain of reactions. The electrons therefore can be “leaked” to a secondary chemical (redox) pathway that is electrically connected to the electrode surface.

Examination of the shape of the polarization curves suggests that the electrochemical device operating with the bioelectrode containing the biofilm of *Chlorella* works under conditions of activation polarization voltage drop due to the small increase of net current with the decrease of the potential. This is usually reflected by a poor kinetics of electron transfer from a catalyst in an electrode surface that hinders the collection of electrons from the electrode. On the other hand, the electrochemical device running with the bioanode containing the biofilm of *Synechococcus* works under conditions of Ohmic polarization. Voltage drop due to the quasi-linear increase of current with the decrease of voltage, means that the kinetics of electron transfer is not a limiting factor for this bioelectrode. In both cases, the voltage drop due to the mass transfer limitation doesn’t appear to play a role within the performance of these bioelectrodes. This is expected for a biocatalyst in the form of biofilm adsorbed in the surface of the electrode containing the active chemicals that may react right away in the surface of the electrode, i.e. not diffusing from the solution to the electrode surface.

McCormick *et al*.^38^ recently presented a very interesting discussion regarding the state-of-art on the current development in BPV systems. The authors discussed the possible mechanisms for the electron transfer involving photosynthetic microorganisms adsorbed in the surface of the electrode, oxidation of end-chemicals produced as metabolites, electrochemical cycling of endogenous lipid-soluble and insoluble natural mediators, direct electron transfer through available surface redox proteins and finally direct contact by conductive nanostructures produced by the cells in the biofilm with the electrode surface^38^. Considering the evidences obtained from this work, we may speculate that the possible mechanisms occurring with the two types of microorganisms working as the bioanodes should be direct electron transfer through redox reactions happening between surface proteins present in the structure of these cells, or possibly through conductive electron transfer structures, such as pili or nanowires. Since the last two proposed structures were never observed in these two types of microorganisms, this possibility is disregarded. The fact that the polarization drop effect in the two microorganisms studied is different suggests that either the mechanism responsible for the electrochemical reactions is different, or the nature of the microorganisms play an important role in the bioelectrochemical process. In fact, *Chlorella* being an eukaryotic algae with a cell wall, presents an organized, and slightly compartmentalized structure, while *Synechococcus* as a prokaryote presents all the intracellular components scattered in the cytoplasm. This peculiarity gives extra mobility to the redox components present inside the cell, which can experience the electrode potential with higher intensity, leading to an increase in the probability to reach and react with the electrode surface.

Examination of the shape of the curves presented in Fig. 5 gives a comprehensive vision of the bioelectrochemical phenomena happening within the biofilm/electrode interface. In both cases, the curves presenting the measurements of current and maximum rate of electron transfer rate seem to be almost mirror imaged. Even though the parameters were obtained from completely different techniques, electrical current measured through the connection with a potentiostat, and the maximum rate of electron transfer rate measured through the PAM fluorescence measurement, there seems to be a direct relationship in the two values, since the increase on the normalized current collected by the bioanode is coupled to the decrease on the normalized maximum rate of electron transfer rate. As mentioned earlier, a correlation coefficient of −0.76 and −0.79 for the values of current density and rETR_m_ for *Chlorella* and *Synechococcus* bioelectrodes, respectively. A value of −1 would represent a perfect correlation coefficient, where every single electron “lost” from the photosynthetic pathway would be given to the electrode for the generation of electricity. The fact that the correlation coefficient in both cases is not close to −1 suggests that this process of electron transfer does not happen directly. This is expected considering that the photosynthetic mechanism is happening at the thylakoid membrane, which is located inside the chloroplast for *Chlorella* and within the cell interior for *Synechococcus*^39^. These two biological structures isolate the internal mechanism from the outside, and therefore the electrons are not coming directly from the photosynthetic pathway, but possibly from a side mechanism connected to the photosynthesis process.

Although the results obtained in this work are suggesting direct correlation between the electrical current generated by the electrochemical device with some of the biochemical redox processes happening on the cells present in the biofilm, the true source of the electrons responsible for the generation of electrical current in both *Chlorella* and *Synechococcus* cannot be confirmed. In spite of that, we believe that we have accomplished a remarkable step towards the comprehension of the fundamental bioelectrochemical processes involving the interface between the biochemical reactions happening within the microorganisms in the biofilm and the electrode reactions involving the transfer of electrons with the generation of electricity. We have reported here a novel tool towards understanding the various fundamental biochemical processes that occur at the electrode and biofilm interface. This is crucial to comprehend the transfer of electrons that generates electricity. The comprehension of these fundamentals is an indispensable factor necessary to overcome the low performance in the generation of electricity that BPVs present.

## Methods

### Growth of algal biofilms on ITO anodes and chlorophyll a (Chl-a) extraction

Two strains obtained from the University of Malaya Algae Culture Center (UMACC), a Cyanophyte *Synechococcus elongatus* UMACC 105 and a Chlorophyte *Chlorella* sp. UMACC 313 (hereafter *Synechococcus* and *Chlorella*, respectively) were used for the bioelectrochemical as well as fluorescence experiments. To obtain the algal biofilms, 100 ml of an exponential phase culture of OD_620nm_ = 0.8 was placed into a 200 ml autoclaved glass staining jar. Indium Tin Oxide (ITO) coated glass slides with diameter 4.4 cm (UQG Optics Cambridge, UK) were placed inside the staining jar with the algal culture, and transferred into an incubator at 24 °C illuminated by cool white fluorescent lamps (30 μmol photons m^−2^ s^−1^) in a 12:12 hour light-dark cycle to allow for the algal biofilms to form on the top of the slides.

The biofilms were removed by washing using jets of distilled water from a pipette, into a sterile beaker to extract the biomass for determination of Chl-a content. Algae cells were then harvested by millipore filtration using filter paper (Whatman GF/C, 0.45 μm) and the Chl-a of the strains were extracted using acetone. The Chl-a concentration was determined using spectrophotometric method^40^. Algal culture collected on a glass-fibre filter paper (Whatman GF/C, 0.45 μm) was mashed using a hand-homogenizer with 10 mL of analytical grade 100% acetone. The samples were then kept in a freezer (4 °C) for 24 hours before being centrifuged (3,000 rpm for 10 minutes at 4 °C). Absorption of the supernatant was measured at 630 nm (OD_630_), 645 nm (OD_645_) and 665 nm (OD_665_). The Chl-a concentration ([Chl-a] in mg.m^−3^) was calculated, considering the volume of acetone used for extraction (V_a_) and the volume of culture (V_c_) using the equation (1):


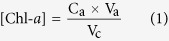


where,


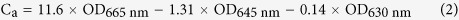


### Pulse Amplitude Modulation (PAM) fluorometer measurement

Photosynthetic parameters were obtained fluorometrically using a Diving-PAM (Walz, Germany), following the protocol discussed in the literature^41^,^42^. Rapid light curves (RLC) were obtained under software control (Wincontrol, Walz). Initially the device was kept in complete darkness for 15 minutes, and thenceforth, red light emitting diodes (LEDs) provided the actinic light used in the RLC at the levels of 0, 33, 96, 186, 291, 425, 576, 835 and 1114 μmol photons m^−2^s^−1^. The RLCs generated for both algal strains showed that maximum relative electron transport rate was achieved at 576 μmol photons m^−2^s^−1^ and started to decrease following the increase of PAR intensity (See Supplementary Tables S1 to S10). Furthermore, a range of saturating pulse had been applied (576 μmol photons m^−2^s^−1^ to 2010 μmol photons m^−2^s^−1^), and this again showed a similar result. Thus, 576 μmol photons m^−2^s^−1^ was selected as the saturating pulse for this study. The biofilm of each ITO slide on day 7 was exposed to each light level for 10 seconds, while the saturation pulse was applied for 1 second^43^.

F_v_/F_m_ was obtained by calculating the variable fluorescence (F_v_), through the measurement of the minimum fluorescence for the dark-adapted cells (F_0_), and the maximum fluorescence obtained after the first saturation pulse (F_m_):


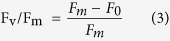


Maximum light utilisation coefficient is determined from the initial slope of RLC termed “alpha” (α)^21^. Light saturation coefficient (E_k_) is obtained from the curve fitting model^24^. The value to be obtained is the interception point of the alpha (α) value with the maximum photosynthetic rate (rETR_m_) and calculated using equation (4).


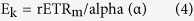


Relative electron transport rate or rETR was calculated by multiplying the irradiance by the quantum yield measured at the end of each actinic light interval. The calculation of the rETR can therefore be made using equation (5)^44^.


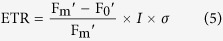


Equation (5) presents the contribution of the three terms for the calculation of the absolute rate of electron transfer. The first term 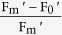 is related to the quantum yield measured at each condition of actinic light given by the fraction of open reaction centers available. The superscript indicates the measurement done with within light, instead of dark-adapted environment. Second term *I* is the irradiance in terms of photon flux (in μmol photons m^−2^s^−1^) from which the photosynthetic material is exposed to. The last term *σ* is the coefficient of light absorption, and it is used as a correction for the second term when the sample is situated in a medium with low transparency, or when the effect of depth plays an important role within the light availability. For experiments performed under thin biofilms, and with artificial medium, the coefficient of light absorption can be ignored. For this case, the calculation is considered to be made for a relative electron transfer rate or rETR rather than just ETR^45^.

### Electrochemical setup and measurement

In concomitance to the fluorescence measurements, the ITO containing the algal biofilm was inserted into the bottom of an electrochemical device, and electrically connected to serve as a bioanode through a pin made of tin touching the surface of the ITO from the top. The connection with the pin was isolated from the solution to avoid corrosion or any other electrochemical reaction that can interfere during the experiment. A disc with 4.4 cm diameter of 40% platinum on carbon paper (FuelCellEarth, USA) was used as the cathode, and connected to the top of the device. A small hole with 1 cm diameter was made in the center of the cathode to allow illumination from the top of the device. The cathode was connected as the working electrode, while the bioanode was connected as the counter and reference electrode in a potentiostat/galvanostat/ZRA “The Reference 600™” (Gamry Instruments, USA). Voltage measured in the electrochemical cell was therefore regarded as the potential difference between the cathode versus the bioanode. A potentiostat/galvanostat was used to apply a constant potential difference across the device. This allowed a range of voltages to be applied to the bioanaode surface and to study the impact of increasing flow of current across the device and its inherent effects on the PAM measurements for the biofilm.

Open circuit potential (OCP) of the electrochemical cell was measured under irradiance for 10 minutes, which is the time necessary for the fluorescence equipment to be stabilized. The electrochemical cell was submitted to steps of chronoamperometry in fixed values of cell voltage between cathode and anode, while the PAM experiments were performed. Values of cell voltage used were; OCP, 200, 150, 100 and 50 mV, and the values of transient current obtained were considered to be the average of the last 100 points of the curve.

This procedure was carried out with biofilms containing *Chlorella* and *Synechococcus* respectively. All experiments were conducted in triplicates, and the results represent the mean value with the error bars representing its repeatability.

## Additional Information

**How to cite this article**: Ciniciato, G. P. M. K. *et al*. Investigating the association between photosynthetic efficiency and generation of biophotoelectricity in autotrophic microbial fuel cells. *Sci. Rep.* **6**, 31193; doi: 10.1038/srep31193 (2016).

## Supplementary Material

Supplementary Information

## Figures and Tables

**Table 1:** Statistical comparison of PAM data of the two algal strains, *Chlorella* sp. and *Synechococcus elongatus*, subjected to cell voltages of 50, 100, 150, 200, 240; data as means ± S.D. (n = 3).

Algae Strains	Cell Voltage(mv)	Fv/Fm	Alpha(α)	rETR_max_ (μ mol electrons m^−2^s^−1^)	E_k_ (μmol photons m^−2^s^−1^)
*Chlorella sp.* (UMACC313)	50	0.60 ± 0.01^a^	0.56 ± 0.01^ab^	64.37 ± 3.11^ab^	114.18 ± 4.329^a^
	100	0.60 ± 0.01^a^	0.57 ± 0.01^ab^	69.80 ± 5.58^a^	121.50 ± 9.045^a^
	150	0.60 ± 0.01^a^	0.58 ± 0.02^ab^	71.39 ± 7.30^a^	124.32 ± 15.24^a^
	200	0.61 ± 0.01^a^	0.59 ± 0.02^a^	71.89 ± 3.94^a^	122.37 ± 10.15^a^
	240	0.62 ± 0.01^a^	0.59 ± 0.01^a^	72.23 ± 4.75^a^	122.29 ± 8.00^a^
*Synechococcus elongatus* (UMACC105)	50	0.35 ± 0.04^b^	0.42 ± 0.01^c^	46.49 ± 2.75^c^	110.39 ± 7.01^a^
	100	0.35 ± 0.04^b^	0.43 ± 0.03^c^	52.26 ± 0.81^bc^	122.70 ± 7.06^a^
	150	0.35 ± 0.03^b^	0.49 ± 0.05^c^	53.06 ± 1.59^bc^	109.92 ± 14.37^a^
	200	0.37 ± 0.03^b^	0.52 ± 0.03^b^	55.86 ± 4.19^bc^	107.92 ± 6.10^a^
	240	0.38 ± 0.03^b^	0.53 ± 0.01^ab^	69.35 ± 4.22^a^	131.59 ± 7.44^a^

Differences between alphabets indicate significant difference between different strains. (ANOVA, Turkey HSD test, p < 0.05).
