# Supplementary material for: Investigating the association between photosynthetic efficiency and generation of biophotoelectricity in autotrophic microbial fuel cells
Source: Sci Rep. 2016 Aug 9;6:31193. doi: 10.1038/srep31193 (PMC4977534; doi:10.1038/srep31193)
Supplement: Supplementary Information [file srep31193-s1.pdf]

Supplementary Information

Correspondence and requests for materials should be addressed to Siew Moi Phang (phang@um.edu.my),Vengadesh Periasamy (vengadeshp@um.edu.my) and Kamran Yunus (ky225@cam.ac.uk)

Investigating the association between photosynthetic efficiency and generation of biophotoelectricity in autotrophic microbial fuel cells

Gustavo P.M.K. Ciniciato[1,4], Fong-Lee Ng[1,2], Siew-Moi Phang[1,2]*, Muhammad Musoddiq Jaafar[3], Adrian C. Fisher[4], Kamran Yunus[4]*, Vengadesh Periasamy[3]*

[1]Institute of Ocean and Earth Sciences (IOES), University of Malaya, 50603 Kuala Lumpur, Malaysia

[2]Institute of Biological Sciences, Faculty of Science, University of Malaya, 50603 Kuala Lumpur, Malaysia

[3]Low Dimensional Materials Research Centre (LDMRC), Department of Physics, University of Malaya, 50603 Kuala Lumpur, Malaysia

[4]Department of Chemical Engineering and Biotechnology, University of Cambridge, New Museums Site, Pembroke Street, CB2 3RA Cambridge, United Kingdom

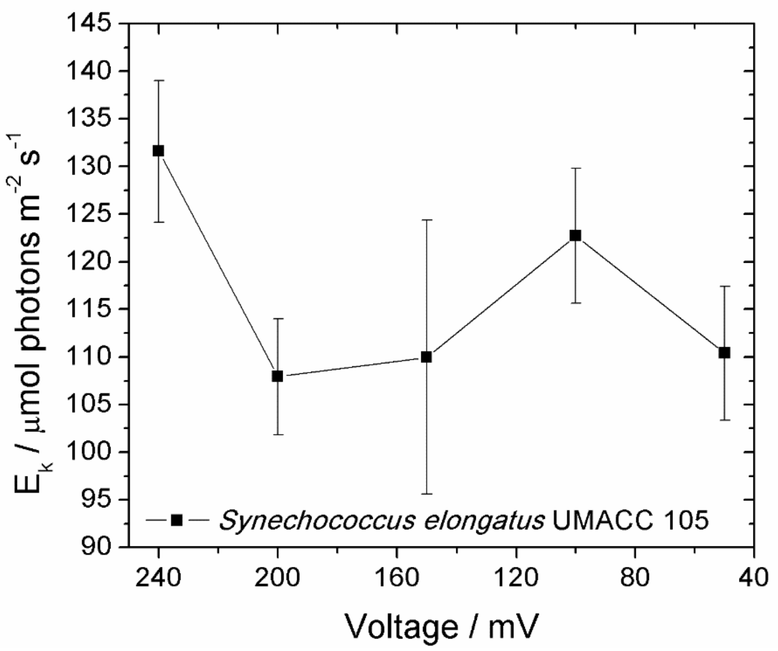

Fig. S1. Effect of the electrochemical cell voltage to the light saturation coefficient E_k in biofilms of *Synechococcus* as bioanodes.

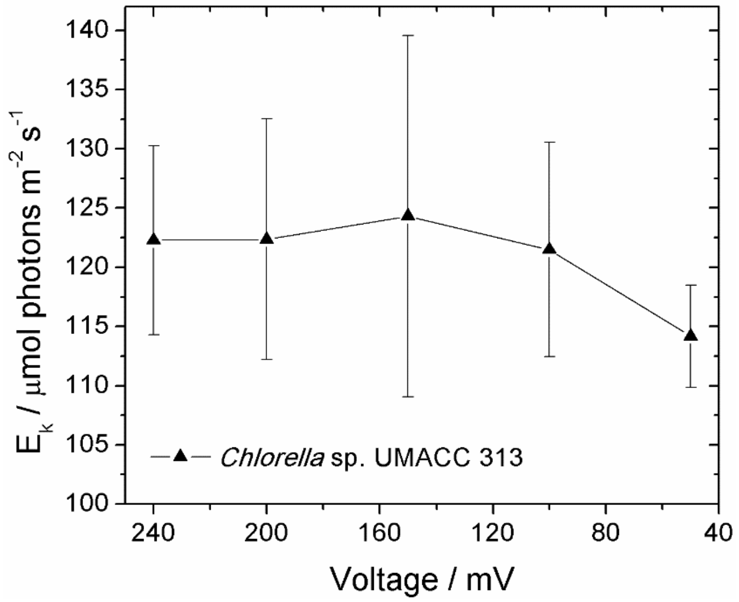

Fig. S2. Effect of the electrochemical cell voltage to the light saturation coefficient E_k in biofilms of *Chlorella* as bioanodes.

Table S1 to S10. Rapid Light Curve for *Chlorella* sp. and *Synechococcus elongatus*, subjected to cell voltages of 240, 200, 150, 100, 50 mV. (n=3, Replicate 1, 2 and 3) PAR: Photosynthetic active radiation (μmol photons $m^{-2}s^{-1}$); rETR: Relative electron transport rate (μ mol electrons $m^{-2}s^{-1}$).

Table S1: Rapid Light Curve for *Chlorella* sp., subjected to cell voltages of 240 mV. (n=3, Replicate 1,2 and 3).

		rETR	
PAR	R1	R2	R3
0	0.00	0.00	0.00
33	16.01	16.07	15.91
96	37.25	38.21	36.77
186	55.99	57.66	54.13
291	67.22	69.26	63.73
425	74.38	77.78	68.00
576	75.46	79.49	70.27
835	71.81	74.32	66.80
1114	69.07	70.18	60.16

Table S2: Rapid Light Curve for *Chlorella* sp., subjected to cell voltages of 200 mV. (n=3, Replicate 1,2 and 3).

		rETR	
PAR	R1	R2	R3
0	0.00	0.00	0.00
33	16.43	15.87	16.04
96	37.92	36.19	36.58
186	58.22	57.66	55.06
291	67.22	65.18	66.93
425	74.80	71.40	73.10
576	77.18	71.42	73.15
835	71.81	68.47	68.47
1114	77.98	64.61	60.16

Table S3: Rapid Light Curve for *Chlorella* sp., subjected to cell voltages of 150 mV. (n=3, Replicate 1,2 and 3).

		rETR	
PAR	R1	R2	R3
0	0.00	0.00	0.00
33	16.43	15.64	15.71
96	37.34	35.04	36.19
186	56.92	52.82	56.54
291	70.71	60.53	67.80
425	77.78	66.30	73.10
576	77.76	65.66	75.46
835	78.49	65.13	68.47
1114	76.87	59.04	61.27

Table S4: Rapid Light Curve for *Chlorella* sp., subjected to cell voltages of 100 mV. (n=3, Replicate 1,2 and 3).

		rETR	
PAR	R1	R2	R3
0	0.00	0.00	0.00
33	16.17	15.51	16.04
96	37.63	35.81	36.10
186	58.22	53.38	54.50
291	70.13	64.89	64.31
425	79.05	68.85	70.13
576	80.64	68.54	72.00
835	77.66	64.30	63.46
1114	64.61	62.38	56.81

Table S5: Rapid Light Curve for *Chlorella* sp., subjected to cell voltages of 50 mV.

(n=3, Replicate 1,2 and 3).

		rETR	
PAR	R1	R2	R3
0	0.00	0.00	0.00
33	16.20	15.54	15.87
96	37.82	36.10	35.52
186	54.31	54.31	55.80
291	66.06	65.77	65.48
425	73.10	73.10	68.85
576	73.73	73.73	70.85
835	78.49	71.81	65.97
1114	62.38	67.95	62.38

Table S6: Rapid Light Curve for *Synechococcus elongatus*, subjected to cell voltages of 240 mV. (n=3, Replicate 1,2 and 3).

	rETR		
PAR	R1	R2	R3
0	0.00	0.00	0.00
33	13.99	11.15	13.60
96	38.59	29.28	38.02
186	56.92	42.78	59.89
291	67.51	48.60	73.62
425	74.38	51.43	83.73
576	73.73	50.11	89.28
835	74.32	50.94	91.85
1114	66.84	52.36	95.80

Table S7: Rapid Light Curve for *Synechococcus elongatus*, subjected to cell voltages of 200 mV. (n=3, Replicate 1,2 and 3).

	rETR		
PAR	R1	R2	R3
0	0.00	0.00	0.00
33	13.56	10.86	13.23
96	36.86	29.28	38.21
186	57.66	43.15	61.38
291	67.51	48.89	73.62
425	71.83	51.85	85.85
576	76.61	52.42	92.16
835	72.65	50.10	90.18
1114	75.75	54.59	91.35

Table S8: Rapid Light Curve for *Synechococcus elongatus*, subjected to cell voltages of 150 mV. (n=3, Replicate 1,2 and 3).

	rETR		
PAR	R1	R2	R3
0	0.00	0.00	0.00
33	12.51	10.43	12.67
96	32.93	28.22	36.10
186	49.85	41.11	57.29
291	57.33	45.69	70.71
425	63.33	49.30	79.05
576	64.51	48.96	83.52
835	63.46	54.28	81.83
1114	59.04	50.13	75.75

Table S9: Rapid Light Curve for *Synechococcus elongatus*, subjected to cell voltages of 100 mV. (n=3, Replicate 1,2 and 3).

		rETR	
PAR	R1	R2	R3
0	0.00	0.00	0.00
33	11.91	10.56	12.84
96	30.91	27.07	36.29
186	47.24	41.29	54.87
291	54.71	46.56	68.68
425	56.95	49.73	73.53
576	61.06	54.14	80.06
835	62.63	52.61	77.66
1114	61.27	51.24	74.64

Table S10: Rapid Light Curve for *Synechococcus elongatus*, subjected to cell voltages of 50 mV. (n=3, Replicate 1,2 and 3).

		rETR	
PAR	R1	R2	R3
0	0.00	0.00	0.00
33	12.24	9.74	12.74
96	33.02	28.51	35.14
186	51.34	42.41	54.50
291	57.91	44.81	66.64
425	63.75	46.75	73.10
576	63.94	47.23	76.03
835	68.47	47.60	77.66
1114	60.16	54.59	75.75
